# Vaccine Dispensing in a Section of the Private Healthcare Sector in South Africa (2017–2021)

**DOI:** 10.3390/ijerph22091329

**Published:** 2025-08-26

**Authors:** Ilse Truter, Johan Hugo, Hank Smith, Shailav Bansal, Alykhan Vira

**Affiliations:** 1 Drug Utilization Research Unit (DURU), Department of Pharmacy, Faculty of Health Sciences, Nelson Mandela University, P.O. Box 77000, Gqeberha 6031, South Africa; 2 Department of Statistics, Nelson Mandela University, P.O. Box 77000, Gqeberha 6031, South Africa; johan.hugo@mandela.ac.za; 3 Quantium Health South Africa, We Work Building, 173 Oxford Road, Rosebank, Johannesburg 2196, South Africa; 4 Quantium Health India, 1 Waverock Building, APIIC Nanakramguda, Serilingampally, Hyderabad 500032, Telangana, India; shailav.bansal@quantium.co.in

**Keywords:** coronavirus disease 2019 (COVID-19), dispensing, pharmacoepidemiology, South Africa, vaccine

## Abstract

The coronavirus disease 2019 (COVID-19) pandemic has put a renewed focus on the value of vaccines in combatting potentially life-threatening diseases. The primary aim was to conduct a longitudinal study on the dispensing patterns of vaccines (from 2017 to 2021) in a section of the private healthcare sector in South Africa. A descriptive cross-sectional pharmacoepidemiological study on health insurance data covering 5 years was conducted. The study included all vaccines available on the South African market (childhood, adult, travel, and other vaccines). The study population consisted of 3.8 million individuals. The descriptive statistics were calculated. The vaccine-dispensing patterns were distinctly different in 2021 compared to the preceding four years. The COVID-19 vaccine was introduced in 2021 in South Africa. Although the total number of medical insurance scheme members stayed relatively constant, the number of vaccine claims increased approximately seven-fold in 2021 compared to the average for the preceding four years (2017 to 2020). The tetanus and pneumococcal vaccines were the most dispensed bacterial vaccines, whilst the influenza and COVID-19 vaccines were the most dispensed viral vaccines. COVID-19 vaccines accounted for 55.74% of all vaccines dispensed over the 5 years, and for 85.70% of the vaccines dispensed in 2021. An increase in the number of bacterial vaccines dispensed was observed towards the middle of 2020, which was attributed to the pneumococcal vaccine. Pneumococcal vaccines were administered during the COVID-19 pandemic to prevent morbidity and mortality from co-/secondary infections and superinfections. Similar ongoing studies on vaccine-dispensing patterns in the post-COVID-19 era are necessary, especially since the outbreak of various vaccine-preventable diseases has recently been observed.

## 1. Introduction

Vaccination is one of the most cost-effective healthcare interventions [1]. The public health and socioeconomic benefits of vaccination gave rise to the World Health Organization (WHO) launching the Expanded Programme on Immunisation (EPI) in 1974, as the first ever global health effort to promote and ensure universal access to vaccines for all children [2]. The programme was also introduced in South Africa. In 1995, the national Expanded Programme on Immunisation in South Africa (EPI-SA), was formed through the unification of all immunisation services in the country [3]. The immunisation landscape in South Africa has developed and expanded since the establishment of EPI-SA, evidenced by the evolution of the vaccination schedule and the establishment of the National Advisory Group on Immunisation (NAGI) in 1993 to advise the National Department of Health (NDoH) on issues related to vaccination [4].

The first six vaccines introduced into the EPI-SA were for diphtheria, tetanus, and pertussis (DTP), measles, polio, and the Bacillus Calmette–Guérin (BCG) vaccine against tuberculosis [5]. As the EPI-SA evolved, new vaccines were introduced, such as the hepatitis B vaccine in 1995 and Haemophilus influenzae type B vaccine in 1999. Since then, the pneumococcal vaccine (PCV) and rotavirus vaccine were introduced in 2009, while the human papillomavirus (HPV) vaccine was introduced in 2014 [4].

Routine immunisation services in South Africa are provided at no cost through the EPI-SA [2,6] to all children in the country. However, parents of children who belong to a private medical insurance scheme may opt to have their children vaccinated through the private healthcare sector. The National Department of Health formulates the policies, procedures, and guidelines to support routine immunisation service implementation across the country through the EPI-SA [3]. Whether the immunisation service is provided in the public or private healthcare sector, the EPI-SA schedule remains the backbone of the childhood immunisations that are required in South Africa.

The Expanded Programme on Immunisation (EPI) in South Africa provides free vaccinations to children, while the private sector offers additional vaccinations and convenience. The EPI schedule focuses on essential vaccines such as vaccines for polio, measles, and hepatitis B, while private practices may offer additional vaccines for chickenpox, meningitis, and Hepatitis A. Both public and private sectors aim to fully immunise children by one year of age, with the private sector often providing a wider range of options and a more personalised service. The vaccination schedules are available at the following websites:

- https://knowledgehub.health.gov.za/system/files/2024-05/VACCINE%20HESITANCY%20WEBINAR_OVERVIEW%20OF%20EPI_MAY_2024_FINAL.pdf (accessed on 15 August 2025) (government/public sector), and
- https://www.amayeza-info.co.za/wp-content/uploads/2025/03/2.-2025-Childhood-vaccine-scheduleN.pdf (accessed on 15 August 2025) (private sector).

In summary, the vaccine schedules are as follows:

Public (EPI-SA) Schedule (free of charge at government clinics):

- Birth: Bacillus Calmette–Guérin (BCG), Oral Polio Vaccine (OPV-0);
- 6 weeks: Oral Polio Vaccine (OPV-1), Rotavirus Vaccine (RV-1), Pneumococcal Conjugate Vaccine (PCV-1), Hexavalent Vaccine (DTaP-IPV-HBV-Hib)-1;
- 10 weeks: Hexavalent Vaccine (DTaP-IPV-HBV-Hib)-2;
- 14 weeks: Rotavirus Vaccine (RV-2), Pneumococcal Conjugate Vaccine (PCV-2), Hexavalent Vaccine (DTaP-IPV-HBV-Hib)-3;
- 6 months: Measles-Rubella Vaccine (MR-1);
- 9 months: Pneumococcal Conjugate Vaccine (PCV-3);
- 12 months: Measles-Rubella Vaccine (MR-2);
- 18 months: Hexavalent Vaccine (DTaP-IPV-HBV-Hib)-4;
- 6 years: Tetanus diphtheria, acellular Pertussis Vaccine (TdaP-1);
- 9–14 years: Human Papilloma Virus Vaccine (HPV) 1 + 2;
- 12 years: Tetanus diphtheria, acellular Pertussis Vaccine (TdaP-2).

Private schedule (additional/optional):

- 6 months: Influenza vaccine (seasonal);
- 9 months: Meningococcal vaccine (Men-A,C,Y,W–1), Pneumococcal Conjugate Vaccine (PCV) booster;
- 12 months: Measles, Mumps, Rubella (MMR), Varicella (chickenpox), Hep A (dose 1), Men-A,C,Y,W-2);
- 18 months: Hexavalent booster, Hep A (dose 2);
- 5–6 years: Diphtheria, Tetanus, acellular pertussis, and Polio vaccine, MMR booster, Varicella booster.

In May 2012, South Africa joined other WHO Member States to endorse the Global Vaccine Action Plan (GVAP) [2]. The GVAP envisioned a world in which all individuals and communities enjoy lives free from vaccine-preventable diseases, with a global target for countries to reach 90% national coverage of all their primary-series vaccines by 2020 [2]. Significant progress has been made through immunisation, resulting in a reduction in the burden of vaccine-preventable illness globally [2], as well as in South Africa, since the introduction of EPI.

The recent pandemic added a new dimension to the history of vaccination. It has been stated that the coronavirus disease 2019 (COVID-19) pandemic has disrupted health and health systems worldwide [7]. COVID-19 has affected virtually all countries in the world, regardless of their economic power, but has presented a particular threat to low- to middle-income countries (LMICs) such as South Africa, as they battle the pandemic alongside pre-existing challenges, such as efforts to control vaccine-preventable diseases and the capacity of health systems to manage the additional burden of COVID-19 [7]. The COVID-19 pandemic has brought a renewed focus on and awareness of the importance of vaccination, but may also have shifted the focus away from other vaccines. Studies on vaccination-dispensing patterns are, therefore, important in order to document and monitor changes.

Not many pharmacoepidemiological studies have been published in the scientific literature on the vaccine-dispensing patterns in South Africa. Pharmacoepidemiology is the study of the use and effects of medicines in large groups of people [8]. Large databases make such studies possible. These studies are important because they not only quantify the number and types of vaccines, but also provide epidemiological data, and can, in time, be used to predict, monitor, and investigate the magnitude of the outbreaks of vaccine preventable diseases.

From a big data perceptive, this descriptive pharmacoepidemiological study addressed the following research question: how did the vaccine-dispensing patterns look like during the years 2017 to 2021, taking into account that the COVID-19 pandemic may have influenced the dispensing patterns during the last two years of the study period?

The primary aim of the study was, therefore, to conduct a pharmacoepidemiological study on the dispensing patterns of vaccines in a section of the private healthcare sector in South Africa (including the EPI-SA vaccines, other adult vaccines, travel vaccines, and COVID-19 vaccines) for the period 2017 to 2021, which serves as a baseline study with which to compare future longitudinal studies. The objectives of the study were as follows:

- To identify the general trends in vaccine claims;
- To determine the demographic distributions;
- To determine the geographical variations;
- To report on the vaccine types dispensed, with a particular focus on the impact of the COVID-19 pandemic on vaccine uptake and dispensing settings.

## 2. Materials and Methods

### 2.1. Study Design

The study design was a descriptive cross-sectional pharmaoepidemiological study based on the analysis of the demographic and claims records of members belonging to medical schemes administered by one of the largest private healthcare insurance providers in South Africa. Data covered a five-year period (2017 to 2021). All patients who were dispensed one or more vaccines were identified using the Anatomical Therapeutic Chemical (ATC) Classification System [9]. All records for vaccines (ATC Group J07) were extracted for analysis. Note that J06 (immune sera and immunoglobulins) were not included in the study.

### 2.2. Study Population

The study population consisted of approximately 3.8 million individuals from 1.8 million households (families) in South Africa. Individuals can belong to various plans that range from budget-friendly to more premium plans, with varying levels of benefits. The term “member” refers to a person who was an insured member of the medical aid scheme at the time of the study, and the word “patient” refers to a member who had submitted one or more claims during the study period (in other words, a patient is a member who claimed (a claimant)).

Population turnover within the insured cohort was considered in the analysis. Although the total number of insured members remained relatively stable, changes in individual membership (entries and exits) had been factored in the analysis. The insured population per year was defined and any coverage estimates were shown for members insured by the scheme at that time. For each study year (2017–2021), the denominators and analyses, therefore, included only individuals (members) who held active insurance coverage during that particular year.

Since the database represents one of the largest private sector databases in South Africa, it is reasonable to assume that the data are generalisable to the broader South African population with private health insurance.

### 2.3. Data Sources

Secondary de-identified demographic and claims data of members belonging to medical schemes administered by one of the largest medical insurance companies in the country (referred to from hereon as “the company”) for the period from 1 January 2017 to 31 December 2022 was extracted from the data warehouse of Quantium Health (Johannesburg, South Africa). Quantium Health is an independent company that provides data analytics and strategic consulting services to the company. For each insured individual, the data contained the following variables: unique study individual identifier, date of birth, gender, and province. For each claim submitted to the administrator for reimbursement of services rendered or items dispensed to an insured individual, the data contained the following variables: a unique study individual identifier; dates for the commencement and completion of the service; a code and description for each service rendered/item dispensed; an ICD-10 (10th revision of the International Statistical Classification of Diseases and Related Health Problems) code [10] for the diagnosis of the condition being treated; a Current Procedural Terminology (CPT) code for the procedure carried out [11]; a National Pharmaceutical Product Index (NAPPI) code [12] for any surgical, medical, or consumable item dispensed; and the amount being claimed.

For each month, the total number of members was used as a denominator to account for the changing membership totals. This allowed for a more accurate representation of claiming patterns.

### 2.4. Data Extraction and Analysis

Snowflake Cloud Solutions^®^ was used to store all scheme data. Structured Query Language (SQL) was used to query and extract data for analysis. Microsoft Excel^®^ was then used to analyse and present data. Descriptive measures include the total number of vaccines (quantity) and the total number of patients relating to claims submitted to the company for each gender, age, and province. A limit on the total number of claims for an individual was set as 40 vaccines per year.

### 2.5. Ethical Considerations

Data for the study was made available as part of Quantium Health’s commitment to supporting research initiatives with broader public health significance. The company does not advise its clients on the clinical treatment of its members. The data was accessed in terms of and under the conditions set out in the agreement between Quantium Health and the company, and a memorandum of understanding between Quantium Health and the study investigators and the university. All the data was provided in a de-identified and aggregated format and the research team had no access to information that would enable the identification of any individual. Data were aggregated in terms of coverage and total vaccines dispensed by age, geographic population, and gender. No data is presented in the manuscript that is on an individual basis and can be used to identify an individual.

The study was also conducted in accordance with the Protection of Personal Information (POPI) Act of South Africa [13]. All findings are presented at an aggregate level and no confidential member, healthcare provider, or scheme information is disclosed. Ethics approval for the use of the database to carry out this study was granted by the Research Ethics Committee (Human) (REC-H) at Nelson Mandela University to conduct research on electronic databases (registration number: H22-HEA-PHA-001). GenAI was not used in this study.

## 3. Results

### 3.1. General Overview of Members and Vaccine Dispensing

The baseline information for vaccines claimed from 2017 to 2021 is given in Table 1 for the number of patients who claimed vaccines, as well as the number of families claiming vaccines during 2021. Table 2 shows the total vaccines claimed and total individuals who claimed for those vaccines indexed from 2017. The total number of medical aid scheme members and families remained relatively constant. There was approximately a seven-fold increase for 2021 in the total number of vaccine doses claimed compared to the average of the preceding four years. This increase was due to the COVID-19 vaccines becoming available and being funded by the scheme. Approximately 65% (65.04%) of all vaccines claimed from 2017 to 2021 were claimed in 2021.

Table 3 shows the proportion of medical aid scheme members and families who received vaccines each year. Just more than half of all members (52.04%) received one or more vaccines in 2021. Two-thirds (66.96%) of families with members belonging to the medical aid scheme claimed one or more vaccines in 2021. It is clear that the dispensing pattern of vaccines in 2021 was distinctly different compared to the preceding four years.

### 3.2. Demographic Information of Patients

Table 4 and Table 5 show the proportion of patients and scheme members by gender over the five-year period, and descriptive statistics such as the average and standard deviation of age by gender, respectively. From Table 4, it can be seen that the proportion of female and male patients has remained relatively constant over the five-year period, with the proportion of female patients being slightly higher than the proportion of male patients. The same can be said for the proportion of female and male scheme members, which remained constant over the same period.

From Table 5, it can be seen that the average age of female and male patients increased in 2021 compared to the preceding four years, likely due to older scheme members being vaccinated against COVID-19, with a higher uptake from older ages compared to younger scheme members who were also eligible. Moreover, the standard deviation in age decreased in 2021, likely due to a large increase in individuals 18 years and older claiming for COVID-19 vaccines, reducing the spread in age seen in the last four years where most of the vaccine uptake was primarily from very young or older members.

### 3.3. Vaccines Dispensed in the Different Age Groups

**Figure 1:**
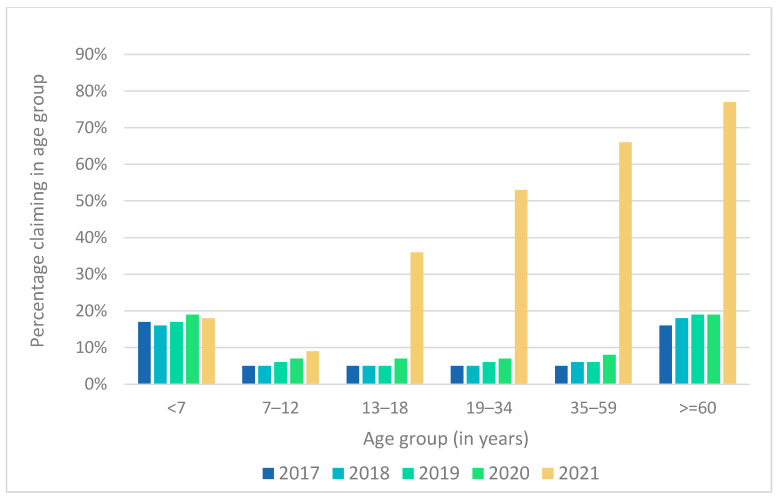
Percentage of patients out of the total number of scheme members in the different age groups who were dispensed vaccines for the period 2017 to 2021.

In Figure 1, the percentages of patients in the different age groups, out of the total number of scheme members in the same age groups, are depicted. From Figure 1, it is clear that there has been a dramatic increase in the percentages of patients who were dispensed vaccines during 2021 in all age groups from 13 years and older. This may be explained in light of the newly developed COVID-19 vaccines, which were rolled out in a three-phase approach, beginning with frontline healthcare workers in Phase 1, followed by essential workers, persons in congregate settings, persons older than 60 years, and persons over 18 years with co-morbidities [14]. Phase 2 of the COVID-19 vaccine roll-out started on 17 May 2021 and continued until October 2021 [14]. Phase 3 focused on persons older than 18 years and started in November 2021 [14]. The 13- to 18-year-old age group started to gain access to COVID-19 vaccinations in August 2021 (most likely those with co-morbidities as well as those working in healthcare settings), with the largest increase for this age group occurring in October 2021 when all 18-year-olds had access.

### 3.4. Dispensing Patterns According to Provinces

**Figure 2:**
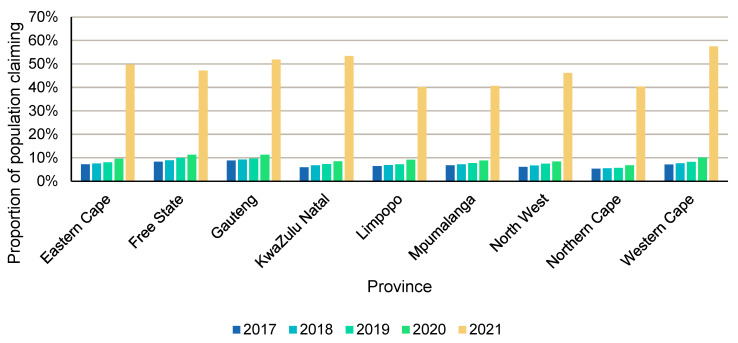
Proportion of patients to whom vaccines were dispensed as a percentage of insurance scheme members residing in the nine provinces for the period 2017 to 2021 *. * Note that, between 2017 and 2021, on average, there was approximately 3.1% of patients who were vaccinated who did not have a location of residence indicated, and these patients are not included in Figure 2.

South Africa consists of nine provinces or regional areas. Prescribing differences are often observed between provinces due to the urban versus rural nature of some of the provinces, as well as the different epidemiological profiles. In Figure 2, the number of patients who were dispensed vaccines as a percentage of insurance scheme members residing in that province is displayed for the period 2017 to 2021. Note that the provincial information shown relates to where the individual who claimed for vaccines resides and not where the vaccine was administered. From Figure 2, it is clear that, during 2021, a dramatic increase in the proportion of patients who were dispensed vaccines can be observed. This dramatic increase observed during 2021 may again be attributed to the roll-out of the newly developed COVID-19 vaccines during this period.

Most vaccines claimed in 2021 were claimed by individuals who reside in the Western Cape, KwaZulu-Natal, and Gauteng provinces. These are the three most populous provinces in the country [15]. Since the initial COVID-19 vaccine roll-out also focused on the major metropolitan areas, this finding was expected. It is also noteworthy that there was a lower overall uptake in vaccines in the Limpopo, Mpumalanga, and the Northern Cape provinces as the proportions of those populations were lower in 2021 compared to those from Western Cape, KwaZulu-Natal, and Gauteng. These three provinces are generally regarded as more rural and are more sparsely populated provinces, without major metropolitan cities and economic hubs such as Cape Town in the Western Cape, Durban in KwaZulu-Natal, and Johannesburg/Pretoria in Gauteng. Prior to 2021, the proportions in provinces such as KwaZulu-Natal, Limpopo, and Mpumalanga were almost comparable, but this pattern changed in 2021 when KwaZulu-Natal had roughly 13% more of their residence population covered compared to the other two provinces.

**Figure 3:**
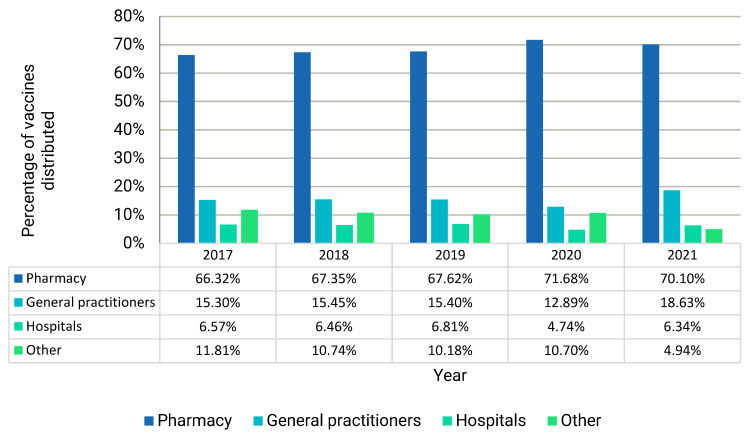
Settings where vaccines were dispensed as a percentage of the total number of vaccines dispensed each year for the period 2017 to 2021.

Figure 3 illustrates the settings where vaccines were dispensed. On average, over the mentioned five-year period, 68.61% of vaccines were dispensed by pharmacies, followed by general practitioners (15.53%), other providers (9.67%), and hospitals (6.18%). Other providers included, for example, auxiliary staff, specialists, and day hospitals. The dispensing patterns observed during 2021 was again distinctly different from the four preceding years, due to the administration of COVID-19 vaccines. South Africans began a roll-out of the Johnson and Johnson COVID-19 vaccine on 17 February 2021, with an initial 80,000 doses administered [16]. Frontline healthcare workers were the first recipients of the vaccine. If the years 2020 and 2021 are not taken into account, on average, 66.10% of vaccines were dispensed by pharmacies, indicating an increase in the administration of vaccines by pharmacies post the start of the pandemic in 2020.

### 3.5. Dispensing of Specific Vaccines According to the ATC Classification System

**Figure 4:**
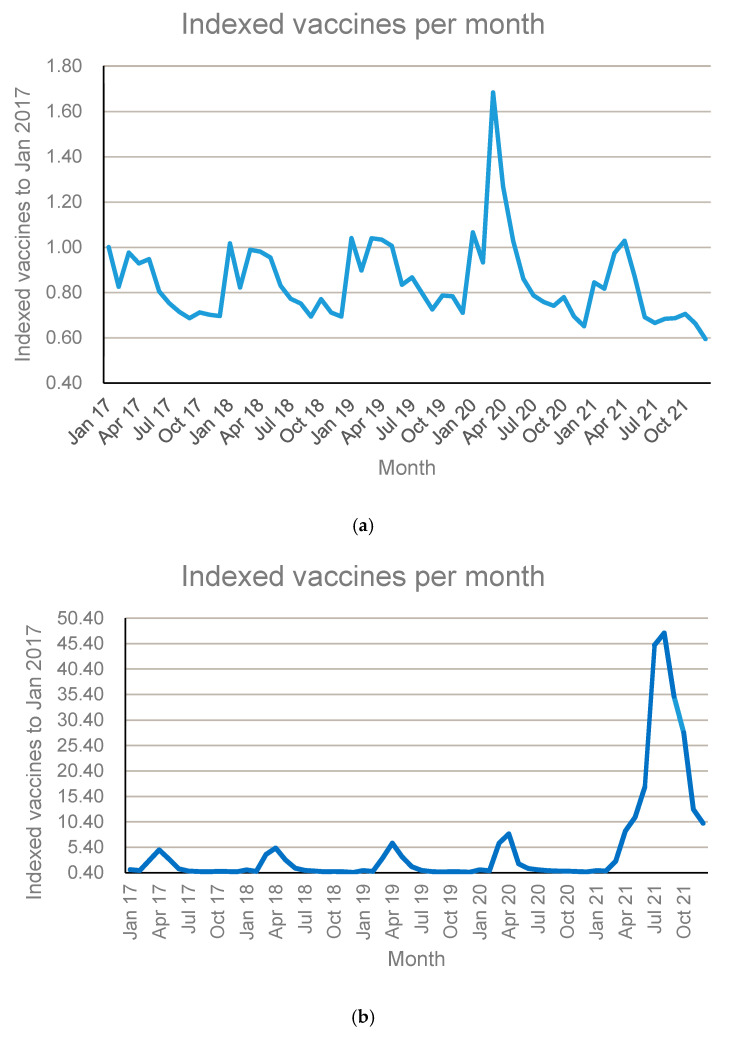

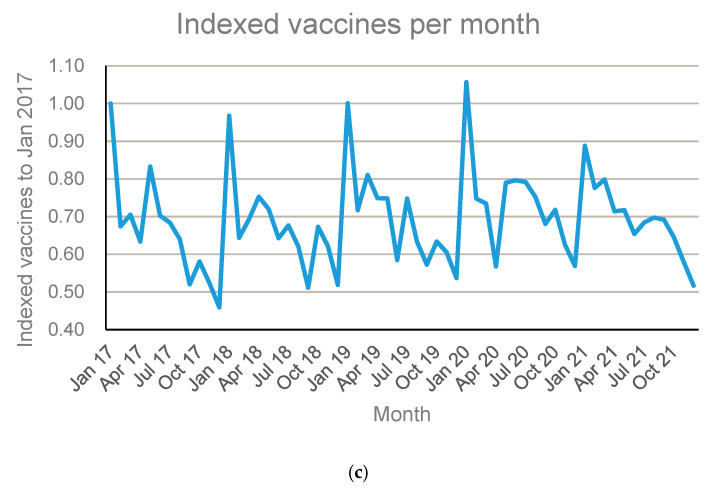
Number of vaccines dispensed per month according to the ATC classification, using 2017 as index base year: (**a**) J07A—Bacterial vaccines; (**b**) J07B—Viral vaccines; and (**c**) J07C—Bacterial and viral vaccines, combined.

According to the ATC Classification System, vaccines are divided into bacterial vaccines, viral vaccines, and combinations of bacterial and viral vaccines, with separate ATC third levels. The total number of vaccines dispensed per month, using 2017 as the index base level year, for the three ATC levels, is illustrated in Figure 4a–c.

The three main categories of vaccines followed a similar dispensing pattern to a previous study conducted using 2015 data [17], with influenza vaccines dominating during March and April of 2020. The annual spike in the dispensing of viral vaccines (J07B) from March to July can be attributed to the time when the annual influenza vaccine becomes available in South Africa. A similar spike in the dispensing of viral vaccines was observed in the 2015 study conducted by Truter [17]. The pattern observed during 2021 was, however, different due to the fact that it was also the time when the COVID-19 vaccine became available in the country.

It must also be mentioned here that the increase observed in the number of bacterial vaccines dispensed towards the middle of 2020 can be attributed to the pneumococcal vaccine (J07AL02). Pneumococcal vaccination was administered during the COVID-19 pandemic to prevent morbidity and mortality from co-/secondary infections and superinfections [18].

**Figure 5:**
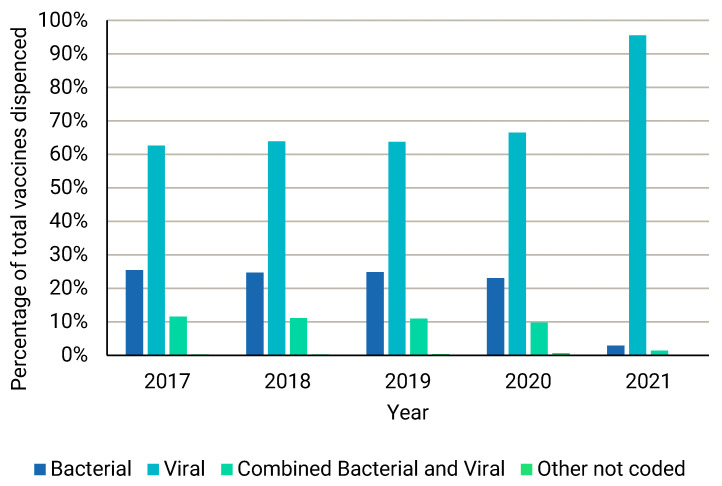
Vaccine types dispensed over the five-year period from 2017 to 2021 as a percentage of the total number of vaccines dispensed.

The vaccine types dispensed over the five-year period from 2017 to 2021, represented as a percentage of the total number of vaccines dispensed, is depicted in Figure 5. From Figure 5, it is clear that, for the mentioned five-year period, the highest percentage of vaccines dispensed was viral vaccines. This is especially true for 2021 as well. During 2021, 95.55% of the total number of vaccines dispensed was viral vaccines.

**Figure 6:**
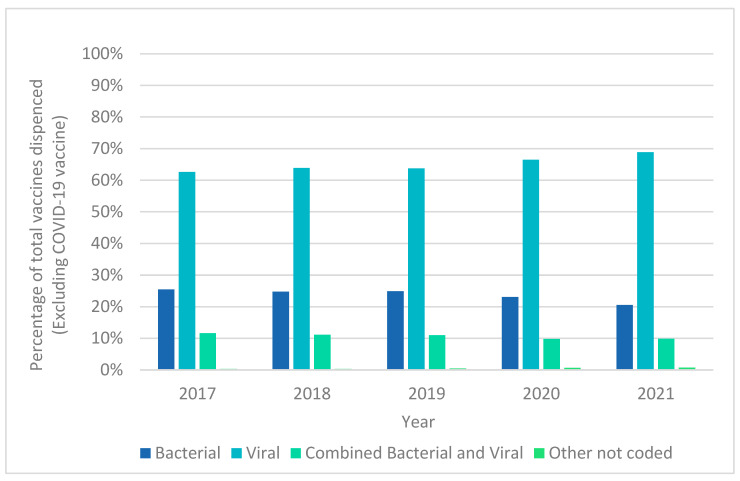
Vaccine types dispensed over the five-year period from 2017 to 2021 as a percentage of the total number of vaccines dispensed, excluding COVID-19 vaccines.

Figure 6 shows the proportion of vaccine types dispensed over the five-year period from 2017 to 2021 as a percentage of the total number of vaccines dispensed, but excluding COVID-19 vaccines.

Table 6 provides a summary of the different vaccines dispensed in the three ATC groups over the five-year period. The tetanus and pneumococcal vaccines were the most dispensed bacterial vaccines in all five years, whilst the influenza and COVID-19 vaccines were the most dispensed viral vaccines, with COVID-19 vaccines accounting for 92.05% of all vaccines dispensed in 2021.

## 4. Discussion

The results were generally in agreement with those of a study on pharmacy dispensing records on 2015 data [17]. In that study, viral vaccines (J07B) also accounted for most of the vaccines (82.7% of volume), followed by bacterial vaccines (J07A), and bacterial and viral vaccines combined. In the current study, viral vaccines accounted for 84.62% of all vaccines, followed by bacterial vaccines (10.45%) and bacterial and viral vaccines combined (4.69%), and 0.23% of vaccines were not coded.

The COVID-19 pandemic has put a renewed focus on the value of vaccines in combatting potentially life-threatening diseases. In the current study, although COVID-19 vaccines dominated vaccine dispensing in 2021, no significant decrease in the proportion of children who have been vaccinated in 2021 was observed. The pandemic has impacted the settings where vaccines are administered. If 2020 and 2021 are not taken into account, on average, 67.10% of vaccines were dispensed by pharmacies, whilst 71.68% and 70.10% of vaccines were dispensed by pharmacies in 2020 and 2021, respectively. More vaccines were, therefore, dispensed by pharmacies in the years following the COVID-19 pandemic. This is in line with the National Department of Health’s efforts to increase the availability of vaccines and also to capacitate community pharmacies to register as vaccination sites [19].

Most vaccines dispensed in 2021 were dispensed to those residing in the three most populous provinces in the country (Western Cape, KwaZulu-Natal, and Gauteng). Overall, there was a lower uptake in vaccines in the Limpopo, Mpumalanga, and the Northern Cape provinces (the proportions of those populations were lower in 2021). These provinces are regarded as more rural provinces, and vaccine access and/or vaccine hesitancy may have played a role. The increase in the number of bacterial vaccines dispensed towards the middle of 2020 is also important to note. This was attributed to the pneumococcal vaccine (J07AL02). Pneumococcal vaccination was promoted and administered during the COVID-19 pandemic to prevent morbidity and mortality from co-/secondary infections and superinfections.

The focus of the study was not on COVID-19 vaccines per se, but the study demonstrated how quickly vaccine-dispensing patterns can change if new vaccines need to be rolled out to the population in a short space of time. Another factor that was evident is the timing of the roll-out, since vaccines could only be made available as they entered the country and decisions had to be made in a short space of time how the vaccines would be distributed between the population (first, health care professionals, and, thereafter, based on age groups) and in the different provinces. The South African Pharmacy Council were pro-active and immediately embarked on a process to upskill more pharmacists to administer vaccines by introducing a supplementary training programme on immunisation and injection technique. The success of this initiative is visible by the fact that pharmacies dispensed the most vaccines in 2021 (the year of the mass COVID-19 vaccine roll-out). This emphasises the crucial role that pharmacists can play in mass vaccination programmes.

There are various lessons that could be learnt from the COVID-19 pandemic vaccine roll-out programme, such as strategies on how to improve equitable access, how the patterns inform healthcare resource allocation, and the differences between provinces. Some provinces (for example, Limpopo and Mpumalanga) had a lower vaccine uptake. Both these provinces are more rural provinces, compared to Gauteng and the Western Cape, for example, which are regarded as urban provinces. Further investigation may indicate geographic disparities or access barriers. Subsequent studies could also investigate the age groups and settings which had a higher uptake to help prioritise resources (vaccines, staff, and cold chain infrastructure) where they are most needed. This information will be relevant for future pandemics or the re-emergence of preventable diseases.

The study had several limitations. Only private sector data was analysed—in other words, vaccines for which a medical insurance company had claims for vaccinations submitted by members. This includes the EPI-SA vaccines. All vaccine claims in the data that were used in this analysis are, therefore, those that were billed to the medical aid scheme, and exclude any vaccines administered through the state or not claimed by the individual. It is also known that, during the pandemic, COVID-19 vaccines were provided free of charge to patients at government facilities and it is possible that patients belonging to the private sector medical aid may have received their vaccine at one of the government vaccination sites, and, hence, will not be captured in this database.

## 5. Conclusions

Follow-up studies on more recent data to investigate vaccines dispensed in the subsequent post-pandemic years may be important, firstly, to investigate the possible post-pandemic vaccine hesitancy, if any, and, secondly, to investigate the threat of two existing public health challenges, namely, polio and measles [7], which have re-emerged in countries where vaccine lapses may have occurred. The results of this study can serve as a baseline for international comparative studies, including comparative studies with other African countries.

## Figures and Tables

**Table 1:** Number of patients, families, and vaccines claimed from 2017 to 2021.

Year	Total Vaccines	Total Vaccine Patients	Total Vaccine Families
2017	464,766	293,464	221,213
2018	489,858	319,758	238,850
2019	512,799	339,863	253,128
2020	592,169	393,496	268,129
2021	3,831,794	1,980,538	1,270,080

**Table 2:** Baseline information of vaccines claimed from 2017 to 2021.

Baseline Information	Year *				
	2017 *	2018	2019	2020	2021
Total vaccine doses claimed	1.00	1.05	1.10	1.27	8.24
Total vaccine patients	1.00	1.09	1.16	1.34	6.75
Total vaccine claiming families	1.00	1.08	1.14	1.21	5.74
Total scheme members	1.00	1.02	1.03	1.02	1.02
Total scheme families	1.00	1.03	1.04	1.03	1.06

* 2017 was used as the index base year.

**Table 3:** Proportion of scheme members and families who received vaccines over the five-year period.

Year	Proportion of Scheme Members Who Claimed for Vaccines	Proportion of Scheme Families Who Claimed for Vaccines
2017	7.89%	12.37%
2018	8.41%	13.01%
2019	8.86%	13.61%
2020	10.40%	14.58%
2021	52.04%	66.96%

**Table 4:** Proportion of patients and scheme members by gender over the five-year period from 2017 to 2021.

Patient and Member Proportions	Years				
	2017	2018	2019	2020	2021
Proportion of patients claiming for vaccines					
Females	0.53	0.53	0.53	0.54	0.53
Males	0.47	0.47	0.47	0.46	0.47
Both genders	1.00	1.00	1.00	1.00	1.00
Proportion of total scheme members overall					
Females	0.52	0.52	0.52	0.52	0.52
Males	0.48	0.48	0.48	0.48	0.48
Both genders	1.00	1.00	1.00	1.00	1.00

**Table 5:** Ages of patients by gender over the five-year period from 2017 to 2021.

Statistics on Ages of Patients Claiming for Vaccines	Years				
	2017	2018	2019	2020	2021
Average age (in years)					
Females	36.52	38.23	38.63	38.35	43.45
Males	34.45	36.47	37.05	36.72	42.64
Both genders	35.54	37.40	37.89	37.60	43.07
Standard deviation of age (in years)					
Females	27.48	27.03	26.95	25.77	19.49
Males	27.57	27.26	27.44	26.86	19.16
Both genders	27.54	27.16	27.19	26.29	19.34
Median age (in years)					
Females	35.00	37.00	37.00	38.00	42.00
Males	33.00	36.00	37.00	38.00	42.00
Both genders	34.00	37.00	37.00	38.00	42.00

**Table 6:** Vaccines dispensed over the five years as a percentage of the total number of vaccines dispensed.

Vaccine Type	ATC Code	ATC Description	YEAR					
			2017 (n = 464,766)	2018 (n = 489,858)	2019 (n = 512,799)	2020 (n = 592,169)	2021 (n = 3,831,794)	Average
J07A—Bacterial vaccines								
Cholera vaccines	J07AE01	Cholera, inactivated, whole cell	0.01	0.05	0.04	0.00	0.00	0.02
Hemophilus influenzae B vaccines	J07AG01	Hemophilus influenzae B, purified-antigen-conjugated	0.07	0.10	0.11	0.03	0.00	0.06
Meningococcal vaccines	J07AH03	Meningococcus A,C, bivalent purified polysaccharides antigen	0.02	0.01	0.01	0.00	0.00	0.01
	J07AH08	Meningococcus A,C,Y,W-135, tetravalent-purified-polysaccharide-antigen-conjugated	5.27	5.59	5.96	5.09	0.73	4.53
Pertussis vaccines	J07AJ52	Pertussis, purified antigen, combinations with toxoids	0.00	0.01	0.01	0.00	0.01	0.01
Pneumococcal vaccines	J07AL01	Pneumococcus, purified polysaccharides antigen	1.04	1.09	1.22	1.26	0.16	0.95
	J07AL02	Pneumococcus, purified-polysaccharide-antigen-conjugated	7.61	6.98	6.67	8.84	0.87	6.19
Tetanus vaccines	J07AM01	Tetanus toxoid	10.34	9.77	9.48	6.94	1.03	7.51
	J07AM51	Tetanus toxoid, combinations with diphtheria toxoid	0.01	0.01	0.01	0.01	0.00	0.01
Tuberculosis vaccines	J07AN01	Tuberculosis, live-attenuated (BCG vaccine)	0.43	0.50	0.77	0.75	0.10	0.51
Typhoid vaccines	J07AP03	Typhoid, purified polysaccharide antigen	0.66	0.64	0.62	0.14	0.02	0.41
J07B—Viral vaccines								
Influenza vaccines	J07BB02	Influenza, inactivated, split virus or surface antigen	32.94	38.13	39.70	44.45	6.51	32.35
Hepatitis vaccines	J07BC01	Hepatitis B, purified antigen	1.02	0.92	1.12	0.80	0.10	0.79
	J07BC02	Hepatitis A, inactivated, whole virus	3.25	2.49	0.40	4.24	0.70	2.22
	J07BC20	Hepatitis vaccines, combinations	0.97	1.02	1.33	0.35	0.04	0.74
Measles vaccines	J07BD01	Measles, live-attenuated	2.64	1.70	1.27	0.70	0.11	1.28
	J07BD52	Measles, combinations with mumps and rubella, live-attenuated	5.65	6.14	6.26	5.63	0.81	4.90
	J07BD54	Measles, combinations with mumps, rubella, and varicella, live-attenuated	2.14	0.10	0.03	0.03	0.01	0.46
Poliomyelitis vaccines	J07BF03	Poliomyelitis, trivalent, inactivated, whole virus	0.16	0.12	0.13	0.12	0.01	0.11
	J07BF04	Poliomyelitis oral, bivalent, live-attenuated	0.51	0.61	0.79	0.61	0.03	0.51
Rabies vaccines	J07BG01	Rabies, inactivated, whole virus	1.18	1.07	1.39	0.97	0.17	0.96
Rotavirus diarrhoea vaccines	J07BH01	Rotavirus, live-attenuated	2.34	1.93	2.29	2.21	0.31	1.82
	J07BH02	Rota virus, pentavalent, live, reassorted	1.76	1.96	1.21	0.82	0.11	1.17
Rubella vaccines	J07BJ01	Rubella, live-attenuated	0.00	0.00	0.00	0.00	0.00	0.00
Varicella zoster vaccines	J07BK01	Varicella, live-attenuated	4.59	4.47	4.33	3.57	0.54	3.50
	J07BK03	Zoster, purified antigen	0.00	0.00	0.00	0.00	0.00	0.00
Yellow fever vaccines	J07BL01	Yellow fever, live-attenuated	0.99	1.10	1.04	0.21	0.04	0.68
Papillomavirus vaccines	J07BM01	Papillomavirus (human types 6, 11, 16, 18)	2.47	2.11	2.44	1.78	0.35	1.83
Other viral vaccines	J07BX03	COVID-19 vaccines	0.00	0.00	0.00	0.00	85.70	17.14
J07C—Bacterial and viral vaccines, combined								
Bacterial and viral vaccines, combined	J07CA02	Diphtheria–pertussis–poliomyelitis–tetanus	3.06	3.75	3.93	3.48	0.51	2.95
	J07CA05	Diphtheria–hepatitis B–pertussis–tetanus	0.00	0.00	0.00	0.00	0.00	0.00
	J07CA06	Diphtheria–haemophilus influenzae B–pertussis–poliomyelitis–tetanus	8.44	6.78	6.20	6.09	0.85	5.67
	J07CA09	Diphtheria–haemophilus influenzae B–pertussis–poliomyelitis–tetanus–hepatitis B	0.09	0.59	0.82	0.20	0.05	0.35
	J07CA10	Typhoid–hepatitis A	0.01	0.00	0.00	0.00	0.00	0.00
Other vaccines not coded		Other *	0.33	0.27	0.41	0.68	0.12	0.36
		TOTAL	100.00	100.00	100.00	100.00	100.00	100.00

* “Other” included 13,609 vaccines not classified into an ATC 5th level in the data. It included products such as Actacel-Pasteur^®^ 0.5 mL, Serum pentavalent vial (Namibia only) vial and Boostrix^®^ pre-filled syringe 0.5 mL (J07CA), Rabipur^®^ (Section 21 medicine) powder and solvent, Rabivax-S^®^ (Section 21 medicine) vial plus diluent (J07BG), and Zostavax^®^ powder for reconstitution vial (J07BK).

## Data Availability

The dataset presented in this article is not available due to the ethical agreement signed by the university. Requests to access the dataset should be directed to the chairperson of the university’s research ethics committee.

## Abbreviations

The following abbreviations are used in this manuscript:

ATC	Anatomical Therapeutic Chemical
BCG	Bacillus Calmette–Guérin
COVID-19	Coronavirus disease 2019
CPT	Current Procedural Terminology
DTP	Diphtheria, tetanus, and pertussis
EPI	Expanded Programme on Immunisation
EPI-SA	Expanded Programme on Immunisation in South Africa
GAVP	Global Vaccine Action Plan
HPV	Human papillomavirus
ICD-10	10th revision of the International Statistical Classification of Diseases and Related Health Problems
LMIC	Low- to middle-income countries
NAGI	National Advisory Group on Immunisation
NDoH	National Department of Health
PCV	Pneumococcal vaccine
SQL	Structured Query Language
WHO	World Health Organization
